# Editorial: Innovative Models in Bone Biology: What can be Learned From Rare Bone Diseases?

**DOI:** 10.3389/fendo.2022.892799

**Published:** 2022-03-31

**Authors:** Teun J. de Vries, Wim Van Hul, E. Marelise Eekhoff

**Affiliations:** ^1^Department of Periodontology, Academic Centre for Dentistry Amsterdam, University of Amsterdam and Vrije Universiteit, Amsterdam, Netherlands; ^2^Center of Medical Genetics, Antwerp University Hospital, University of Antwerp, Antwerp, Belgium; ^3^Department of Internal Medicine, Section Endocrinology, Amsterdam University Medical Center (Amsterdam UMC), Amsterdam Bone Center, Vrije Universiteit Amsterdam, Amsterdam, Netherlands

**Keywords:** cell models, rare bone diseases, animal models, fibrodysplasia ossificans progressiva (FOP), fibroblast, osteocytes, osteoclasts, genetics

## Introduction

Since the elucidation of the human genome in 2000, all human genes are known. Subsequently, medical science has bloomed in identifying disease-specific causative mutations. For rare bone diseases, pivotal discoveries of causal genes were for instance the *SOST* gene encoding sclerostin for Van Buchem disease and sclerosteosis (1) or *ACVR1* for fibrodysplasia ossificans progressiva (2). For broadening mechanistic insight, such discoveries require animal- and cell-based models, for instance mouse models with knocked-out or induced expression of the mutated gene (3) or induced pluripotent stem cells (4). Such disease-tailored models were at the forefront of mechanistical discoveries and can lead to therapeutical intervention in the near future (5). The current topic with its 10 contributions, hopes to contribute to the new and still remaining challenges in the field of rare bone diseases by identifying current models or by refining suitable and innovative models.

## New Animal Models in Rare Bone Disease Research

Knock-out mice have been available since the early 1990s, soon followed-up by inducible knock-out mice. These developments have turned out to be valuable for elucidating mechanisms in common bone diseases such as periodontitis (6). Brommage and Ohlson have summarized the state-of-the art of mouse models in bone research and their utility for the human equivalent. An impressive 96% (249 out of 260) of genes that were studied in mice, mimicked a known human variant with skeletal anomalies. In the past decade, zebrafish models have come to the forefront as new models for studying rare bone diseases. Tonelli et al. introduce us to the bone biology of zebrafish and demonstrate that this model is relatively easy for manipulating genes, for instance using CRISPR-Cas9 technology, that can be relevant for rare bone diseases.

## Cell-Based Models From Patients With Rare Bone Diseases

To gain mechanistical insight, knowledge of the causative cell type in rare bone diseases should be the starting point for *in vitro* studies. Appropriate cell models to study rare bone diseases could be challenging, but the most appropriate model seems bone cells that are isolated from biopsies from patients. Thus, one could consider *ex vivo* material of bone chips with viable osteocytes still present (Pathak et al.). Osteocytes produce a variety of proteins and signaling molecules such as sclerostin, cathepsin K, Wnts, DKK1, DMP1, IGF1, and RANKL/OPG to regulate osteoblast and osteoclast activity. Various genetic abnormality-associated rare bone diseases are related to disrupted osteocyte functions is the case in Van Buchem’s disease and sclerosteosis, which are related to non-functional sclerostin. Pathak et al. suggest that future research in rare bone diseases could also aim at restoring function of osteocytes. Fibrodysplasia ossificans progressiva is a rare bone disease where bone biopsy-related bone cells cannot be obtained since this could lead to worsening of the disease. Useful alternatives to study osteogenesic aspects, are skin (7) or periodontal ligament fibroblasts, scraped and isolated from extracted teeth (8). Claeys et al. describe the state-of-the art of fibroblast models in bone research. The osteoclast has been entirely neglected in FOP research, a disease with more bone. Schoenmaker et al. have used monocytes isolated from peripheral blood from controls and FOP patients to study the effect of FOP ligand and bone morphogenetic protein (BMP) Activin-A on osteoclast formation. Although no disease specific effect was observed, interestingly, this ligand caused fewer but larger osteoclasts. Therefore, studies aimed at elucidating rare bone disease mechanisms, may also contribute to more fundamental knowledge on the formation of multinucleated cells. Bone marrow derived mesenchymal stem cells (BMSCs) is yet another example of an appropriate cell model for osteogenesis that could be used in rare bone diseases. By manipulating its expression in BMSCs, Liu et al. show an important role for Chordin-like1 in increasing BMP4 driven osteogenesis. In a series of complementary experiments, the relationship between Chordin-like1 and BMP4 was established, culminating in experiments with bone defects and positive effects of Chordin-like1 on bone healing. Mild phenotypes of rare bone diseases may manifest later in life. Norwitz et al. describe a case of a novel LRP5 mutation in a professional runner, who turned out to be osteoporotic at the age of 18. Here, genetics overrules the bone dogma that impact loading improves bone quality. Huybrechts et al. update the current knowledge of Wnt signaling and rare bone disease. The overview of the skeletal and extra-skeletal phenotypes of the different monogenic skeletal disorders were linked to deviations in the WNT signaling pathway.

## New Perspectives

Our era has gradually unveiled mysteries of many rare bone diseases by identifying genes, ligands, and pathways that are causative. Nevertheless, despite this tremendous progress, one could also step back and take the liberty to place an old disease into a new framework. Pignolo et al. have done this for FOP, by comparing clinical symptoms that coincide between progeria, or expedited aging, and FOP. Progeroid features that may primarily be associated with mutations in *ACVR1* include osteoarthritis, hearing loss, alopecia, subcutaneous lipodystrophy, myelination defects, heightened inflammation, menstrual abnormalities, and perhaps nephrolithiasis.

For finding the genetical cause of rare bone diseases, technological innovations in the field of sequencing, such as massively parallel sequencing (MPS), have broad potential applications. MacInernery-Leo and Duncan describe the historical development of finding causative mutations and demonstrate that MPS has high potential for future findings of new genetic insight in rare bone diseases. This technique speeds up discovery of causative genes from years to weeks.

## Conclusion

The 10 contributions to this topic on innovative models for rare bone diseases have demonstrated the progress of rare bone disease models in research. For future research, a lot can be expected from CRISPR-Cas9 restored or induced gene function, in combination with induced pluripotent stem cells, since this could build reliable and clean read-out models, where only the mutation is induced or restored. Technological advances in speed of sequencing will faster and more accurately than ever identify novel mutations. Together with our increased biological understanding of the various rare bone diseases, it can be anticipated that clinicians will have more comprehensive guidelines for intervention for the benefit of the patient. In this way, it can be foreseen that quality of life will increase of patients with rare bone diseases.

## Author Contributions

TV initiated writing, WH and EE contributed to editing the draft text. All authors contributed to the article and approved the submitted version.

## Conflict of Interest

The authors declare that the research was conducted in the absence of any commercial or financial relationships that could be construed as a potential conflict of interest.

## Publisher’s Note

All claims expressed in this article are solely those of the authors and do not necessarily represent those of their affiliated organizations, or those of the publisher, the editors and the reviewers. Any product that may be evaluated in this article, or claim that may be made by its manufacturer, is not guaranteed or endorsed by the publisher.
